# Challenges in the Repatriation Processes of a Foreign Citizen With Schizophrenia: A Case Report From Tanzania

**DOI:** 10.1155/crps/3483266

**Published:** 2025-02-05

**Authors:** Frank Kiwango

**Affiliations:** ^1^Department of Psychiatry and Mental Health, Kilimanjaro Christian Medical Centre, Moshi P.O. Box 3010, Tanzania; ^2^Faculty of Medicine, Kilimanjaro Christian Medical University College, Moshi P.O. Box 2240, Tanzania; ^3^Department of Psychiatry and Mental Health, Muhimbili University of Health and Allied Sciences, Dar es Salaam P.O. Box 65014, Tanzania

## Abstract

**Background:** Schizophrenia is a chronic mental health disorder characterized by an array of symptoms, leading to impairment of functioning. Many patients with schizophrenia tend to have long-stay hospitalizations due to several factors, one of them being the repatriation process.

**Case Presentation:** I report the case of a 29-year-old male foreign citizen who presented with auditory hallucinations, paranoid delusions, and aggressiveness. The patient had a history of multiple admissions due to poor drug adherence. After being admitted to the psychiatry ward, the patient was improved and ready for discharge after 4 weeks but struggled to remember his relatives' phone numbers. Due to financial constraints and poor support, the repatriation process was delayed for 160 days.

**Conclusion:** The present case highlights the challenges in managing schizophrenia abroad, urging international protocols to streamline the repatriation process and address financial, logistical, and social barriers for improved outcomes.

## 1. Introduction

Schizophrenia is a chronic mental health disorder characterized by a range of symptoms, such as delusions, hallucinations, disorganized speech or behaviors, and negative symptoms [1]. Its chronic nature and early onset make it highly disabling [1, 2]. This results in decreased functioning of a person's daily activities such as work, interpersonal relationships, and self-care [3] and may lead to long-term hospitalizations and frequent readmissions [2]. Recently, the length of stay (LOS) for psychiatric patients with schizophrenia has become a significant concern for researchers, and in sub-Saharan Africa, the LOS varied from 22 to 128 days [4–6]. Factors influencing LOS include delay of repatriation, which is the process of returning an individual from a foreign country to their homeland [7, 8]. Foreign patients face stigmatization due to both their mental illness and being non-native strangers in an unfamiliar country [9–11]. The process is often delayed due to the lack of international protocols for transporting and treating mentally ill patients, the high cost of treatment, and other aspects of the repatriation process [10].

## 2. Case Report

Here, I present a 29-year-old man, the fourth born out of seven siblings, whose both parents deceased when he was a teenager. The patient finished primary school and did not proceed to secondary school due to financial constraints. He was married to a housewife and had two children who were in primary school. The patient worked as a mechanic to earn his living in Thika, Kenya. Since the age of 18 years, he had been treated for schizophrenia with intermuscular (IM) injection of fluphenazine 25 mg monthly and orally distributed chlorpromazine 400 mg twice daily. He reported having multiple admissions due to poor drug adherence because of inadequate financial constraints for buying medications. The patient did not earn enough money in Kenya to support his family. Therefore, he decided to go to Dar-es-salaam, a major city of Tanzania, where he got temporary employment at a garage and worked as an assistant mechanic. During his stay in Dar-es-salaam, the patient lived in the corridors of that garage since he could not afford a rented house. He had been paid a very low salary that could not sustain his daily living. Still, the patient continued to stay in Dar-es-salaam believing that he would get a better job. He reported this condition being very stressful. Seven months after arrival, he complained of hearing voices others could not hear. He could hear people talking to themselves and was suspicious that some of his neighbors and workmates wanted to harm him. This made him physically aggressive which necessitated the good Samaritans to call the police. Officers from the central police station brought the patient to the psychiatry department at Muhimbili National Hospital in Dar-es-salaam. The patient reported not being on any medications since his arrival in Dar-es-salaam 1 year earlier. During arrival, he was conscious, with bilateral wrist wounds due to restraining, but was not febrile, not pale, not jaundiced, not cyanosed, and without lower limb edema. He had stable vital signs where the blood pressure was 124/86 mmHg, pulse rate 78 beats/min, body temperature 36.8 C, and respiratory rate of 19 cycles/min. On mental status examination (MSE), he was unkempt, uncooperative, and aggressive, with third-person auditory hallucinations, and paranoid delusions. Also, he was not oriented to people, place, and time.

The patient was admitted to the psychiatry acute ward and managed with admission protocol, where he was prescribed IM haloperidol 5 mg every half hour for 2 h and IM diazepam 20 mg every 8 h for 24 h. Laboratory investigations including full blood picture, renal and liver function test, malaria rapid test, and random blood glucose were within normal range. During postadmission ward rounds, the patient was calm but expressed auditory hallucinations and paranoid delusions. He also had circumstantial thoughts, poor short- and long-term memory, poor abstract thinking, and poor judgment with poor insight. The patient was transferred to the general male ward where he was treated with oral haloperidol tablets 3 mg twice daily; in addition, he received psychological insight-orientated intervention and attended occupational therapy while continuing with social exploration for tracing relatives. After 4 weeks, the patient had a stable MSE and was fit to be discharged. However, the patient could not remember any phone numbers of his relatives or friends. We continued with the social exploration by integrating with the patient's embassy. The patient continued to stay in the psychiatry ward, receiving his daily medications while waiting to be repatriated to Kenya. However, the repatriation process was delayed for 160 days. The patient was later repatriated to Kenya by his embassy. The reasons for delaying repatriation were failure to pay for treatment which was later waived by the hospital and initial lack of willingness of the embassy to support the patient.

## 3. Discussion

The repatriation process has to be in the patient's best interests. These steps include contacting relevant embassies, arranging interpreters, obtaining psychiatric history, and ensuring continuity of care until the patient is fit to travel [10].

However, the effectiveness of repatriation depends on the timely execution of these steps and the availability of resources. In this case, it emphasizes that the financial constraint was a setback as also seen from other studies [9, 10]. The financial burden of long-term hospitalization includes the costs of inpatient admissions and medications.

Repatriation is often delayed, not only because of financial barriers but also due to the need for careful planning and coordination among various stakeholders, including embassies, medical professionals, and the patients' families. In this case, we could not get in hold of the patient's family due to his inability to remember the phone and other contact numbers. We also failed to access the social worker in Kenya directly from Tanzania.

In addition, there was a lack of international protocols that could be followed if the repatriation process was delayed [10]. In this case, it led to prolonged hospital stays which strained the health care resources and also increased the burden on the patient.

Due to the nature of the disease, the patient had deficits in cognitive domains that made it difficult for him to recall his relatives' whereabouts and phone numbers to easily reach out [3, 7]. This complicated the management and repatriation of the patient.

Lastly, the stigma faced by patients with severe mental illness abroad exacerbates their situation. They are often stigmatized both for their mental health condition and their status as foreigners [9]. This dual stigma can lead to human rights violations and delays in receiving appropriate treatment.

In conclusion, the discussion underscores the multifaceted challenges in managing schizophrenia, particularly for patients abroad. It calls for the development of international protocols to streamline the repatriation process and ensure that patients receive timely and appropriate care. This can be done by developing agreements such as bilateral health treaties among countries that outline the roles of embassies in assisting patients abroad by addressing the financial, logistical, and social barriers to the repatriation process.

## Data Availability

Data sharing is not applicable as no new data were generated.
